# Alveolar Rhabdomyosarcoma With Bone Marrow Metastases and Disseminated Intravascular Coagulation Mimicking Acute Lymphoblastic Leukemia: A Case Report

**DOI:** 10.7759/cureus.88298

**Published:** 2025-07-19

**Authors:** Songlin Chu, Hongbin Zhang, Qian Niu, Liansheng Zhang, Lijuan Li

**Affiliations:** 1 Department of Hematology, The Second Hospital & Clinical Medical School, Lanzhou University, Lanzhou, CHN; 2 Department of Pathology, The Second Hospital & Clinical Medical School, Lanzhou University, Lanzhou, CHN

**Keywords:** acute leukemia, alveolar rhabdomyosarcoma, bone marrow metastases, disseminated intravascular coagulation, misdiagnosis

## Abstract

Rhabdomyosarcoma (RMS) is the most common type of soft tissue sarcoma in children and adolescents, with some patients exhibiting bone marrow involvement. Leukemic presentations of RMS have been documented in the literature. However, distinguishing RMS with bone marrow metastasis from acute leukemia can be challenging due to their overlapping morphological features, particularly in the absence of a characteristic primary mass. In this report, we present a case of alveolar rhabdomyosarcoma (ARMS) that was initially misdiagnosed as acute lymphoblastic leukemia (ALL). After reviewing similar case reports, we propose that the presence of scattered cytoplasmic vacuoles in the blast-like cells of the bone marrow, significant differences in the percentage of blast-like cells between the bone marrow and peripheral blood, and the presence of tumor masses in locations typically associated with RMS may serve as indicators for differentiating RMS that mimics acute leukemia.

## Introduction

Rhabdomyosarcoma (RMS) accounts for approximately 50% of pediatric soft tissue sarcomas but less than 4% of adult cases, with significant differences in histologic types, primary locations, metastatic patterns, and prognoses between these two age groups [1-3]. ARMS, one of the four primary subtypes of RMS, along with embryonal RMS (ERMS), pleomorphic RMS (PRMS), and sclerosing/spindle RMS, represents 20% of pediatric cases and 23% of adult cases [2]. Recently, ARMS has been categorized into fusion-positive and fusion-negative subtypes based on the presence or absence of fusion genes associated with the paired box proteins PAX3 and PAX7, as well as the forkhead box protein O1 (FOXO1). Clinically, ARMS typically occurs in anatomical regions including the extremities, paraspinal areas, perineum, and paranasal sinuses. Approximately 25-30% of cases present with metastatic disease, which primarily disseminates through the lymphatic system and blood vessels, while involvement of the bone marrow is relatively uncommon [1,2,4]. The leukemia-like diffuse infiltration pattern and morphological characteristics exhibited by ARMS tumor cells complicate the differentiation of ARMS from acute leukemia based solely on morphology. This challenge is further compounded by the rarity of ARMS and its predominance in children and adolescents. However, the current MICM (morphology, immunology, cytogenetics, and molecular biology) examination for the accurate diagnosis and prognosis assessment of leukemia includes flow cytometry analysis. This technique has the capacity to identify tumor cells of non-hematopoietic origin, thereby helping to correct potential misdiagnoses. Furthermore, bone marrow or primary mass biopsies, combined with immunohistochemical staining using markers such as MyoD1, myogenin, and desmin, indicative of skeletal muscle differentiation, are essential for confirming the diagnosis. The present case report has two objectives: first, to assist clinicians in recognizing the diagnostic pitfalls of ARMS masquerading as acute leukemia, and second, to provide information for future retrospective analyses.

## Case presentation

A 40-year-old female farmer with no significant medical history presented with multiple swellings on the right side of her neck, each approximately the size of soybeans and exhibiting slight tenderness upon palpation. She reported no accompanying symptoms, such as fever, cough, or night sweats. Initially, she attributed these swellings to fatigue related to her agricultural labor. However, she later sought medical attention at a local clinic, where she was diagnosed with acute lymphadenitis and prescribed empirical penicillin therapy. Despite this treatment, the cervical lymph nodes continued to enlarge progressively and became increasingly painful. Following a referral to our hospital, the patient was admitted to the hematology department with a suspected diagnosis of malignant lymphoma. Table 1 presents the patient's most significant laboratory results upon admission.

**Table 1:** Laboratory findings at admission indicating thrombocytopenia, hypofibrinogenemia, and elevated levels of LDH, D-dimer, FDP, TAT, and PIC. LDH: lactate dehydrogenase, FDP: dibrin degradation product, TAT: thrombin-antithrombin complex, PIC: plasmin-α2-plasmin inhibitor complex

Category	Parameter	Result	Normal Range
Routine blood tests	White blood cell count (WBC)	6.46 × 10^9/L	3.5-9.5 × 10^9^/L
	Neutrophils (%)	75.70%	40-75%
	Lymphocytes (%)	16.00%↓	20-50%
	Monocytes (%)	7.10%	3-10%
	Hemoglobin (HGB)	115 g/L	115-150 g/L
	Platelet count (PLT)	50 × 10^9^/L↓	125-350 × 10^9^/L
Coagulation tests	Prothrombin time (PT)	13.5 sec	9.4-12.5 sec
	Activated partial thromboplastin time (APTT)	28.6 sec	25.4-38.4 sec
	Fibrinogen (FIB)	1.52 g/L↓	2-5 g/L
	D-dimer	68.82 μg/mL↑	<0.50 μg/mL
	Fibrin degradation products (FDPs)	172.48 μg/mL↑	<5 μg/mL
	Thrombin-antithrombin complex (TAT)	83.8 ng/mL↑	<4.0 ng/mL
	Plasmin-α2-plasmin inhibitor complex (PIC)	13.3 μg/mL↑	<0.8 μg/mL
Abnormal serum biochemistry	Aspartate aminotransferase (AST)	53 U/L↑	13-35 U/L
	Gamma-glutamyl transferase (GGT)	105 U/L↑	7-45 U/L
	Uric acid (UA)	690.0 μmol/L↑	155-357 μmol/L
	Lactate dehydrogenase (LDH)	1104 U/L↑	120-250 U/L
	Ferritin	502 ng/mL↑	13-150 ng/mL
	Triglycerides (TG)	4.73 mmol/L↑	0.56-1.70 mmol/L

**Figure 1:**
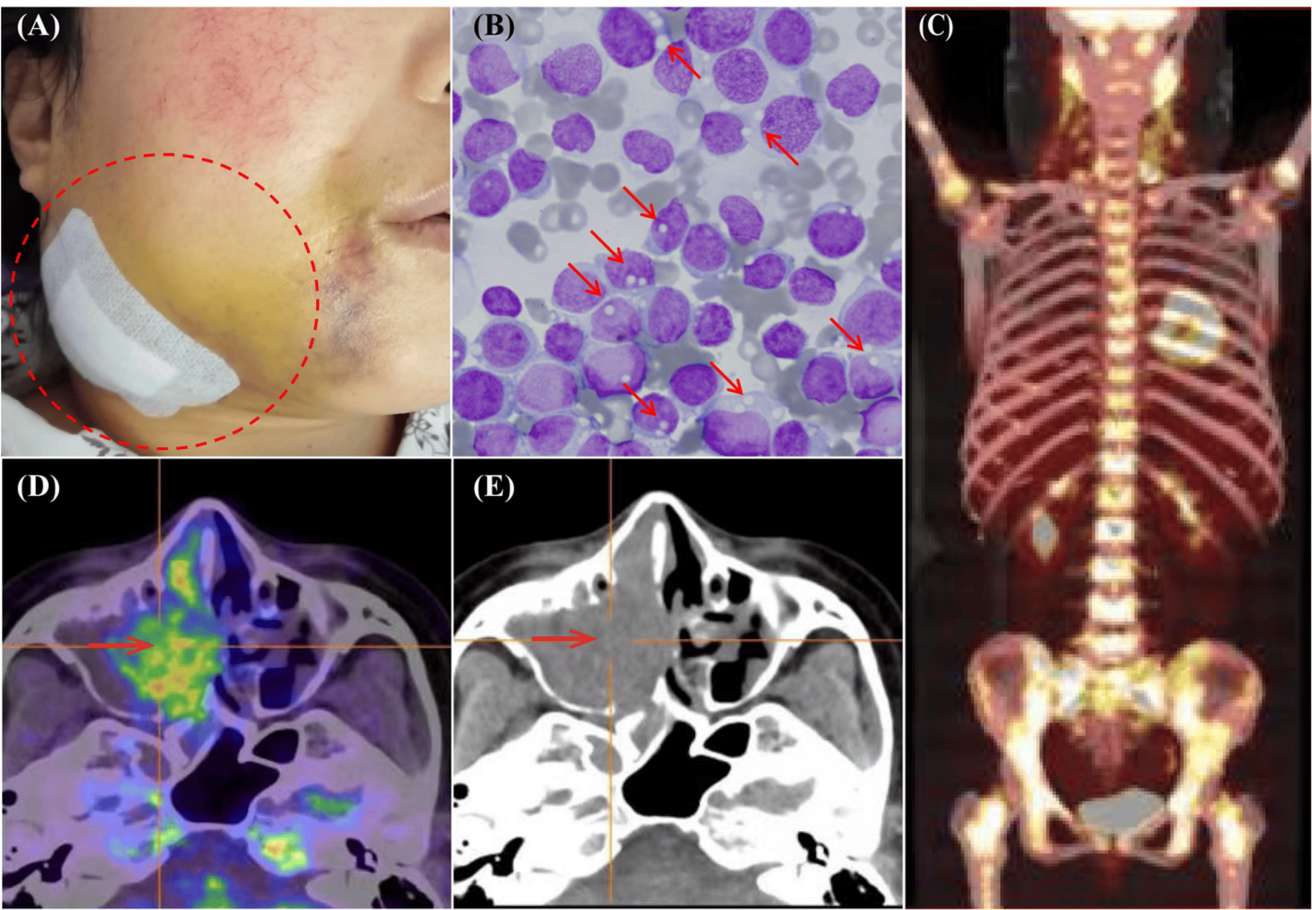
(A) Post-biopsy imaging reveals enlarged cervical lymph nodes (red circle). (B) Bone marrow aspirate demonstrates lymphoblast-like cells (98% cellularity) with non-granular basophilic cytoplasm and scattered vacuoles (red arrow; Wright-Giemsa, ×1000). (C) PET/CT scan shows diffusely increased bone metabolic activity. (D-E) PET/CT images display soft-tissue-density opacities consistent with an infiltrating mass in the right ethmoid sinus, nasal cavity, and maxillary sinus (red arrow).

The patient's laboratory results and clinical features suggested a diagnosis of malignant lymphoma or acute leukemia, which could explain the enlarged cervical lymph nodes (Figure 1A), thrombocytopenia, elevated lactate dehydrogenase levels, and signs of disseminated intravascular coagulation (DIC). A bone marrow aspirate revealed that 98% of the cellularity consisted of lymphoblast-like cells. These cells had non-granular basophilic cytoplasm with scattered vacuoles (Figure 1B) and exhibited negative peroxidase staining. However, only about 1% of these cells were present in the peripheral blood. An 18F-fluorodeoxyglucose positron emission tomography/computed tomography (PET/CT) scan revealed multiple enlarged, hypermetabolic lymph nodes in the bilateral cervical (I-V), parapharyngeal, clavicular, and left axillary regions. In addition, opacities with soft tissue density, indicative of an infiltrating mass, were observed in the right ethmoid sinus, right nasal cavity, and right maxillary sinus. Evidence of soft tissue thickening in the nasopharynx and diffuse enhancement of bone metabolic activity was also noted. The PET-CT findings are highly suggestive of lymphohematopoietic malignancies, with lymphoma being the most likely diagnosis (Figure 1C-1E). On the third day of admission, ALL was diagnosed primarily based on bone marrow morphology and PET-CT scan results. Following Chinese guidelines for the treatment of acute lymphoblastic leukemia, we initiated prednisone acetate therapy prior to induction chemotherapy to reduce the risk of tumor lysis syndrome [5]. Simultaneously, we administered plasma and cryoprecipitate transfusions, along with fibrinogen supplements and low-molecular-weight heparin anticoagulation. We also implemented alkalinization, intravenous hydration, and analgesia as supportive treatment. Concurrently, we conducted flow cytometry, genetic analysis, and chromosomal karyotyping assays on preserved bone marrow specimens.

**Figure 2:**
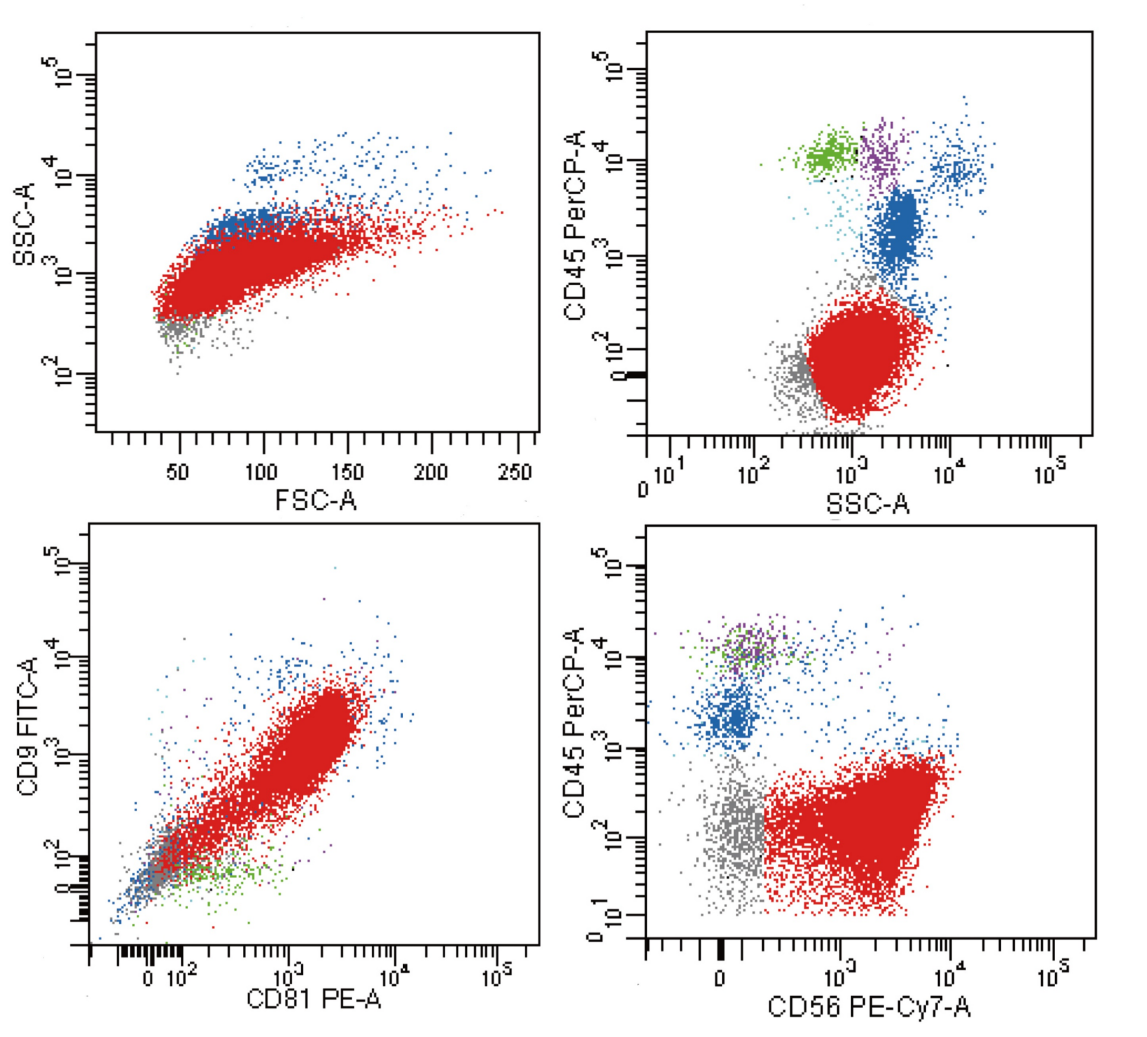
Flow cytometry analysis demonstrated CD45-negative cells (88.9% of nucleated cells) expressing CD9, CD81, and CD56, indicative of nonhematologic origin (highlighted in red).

**Figure 3:**
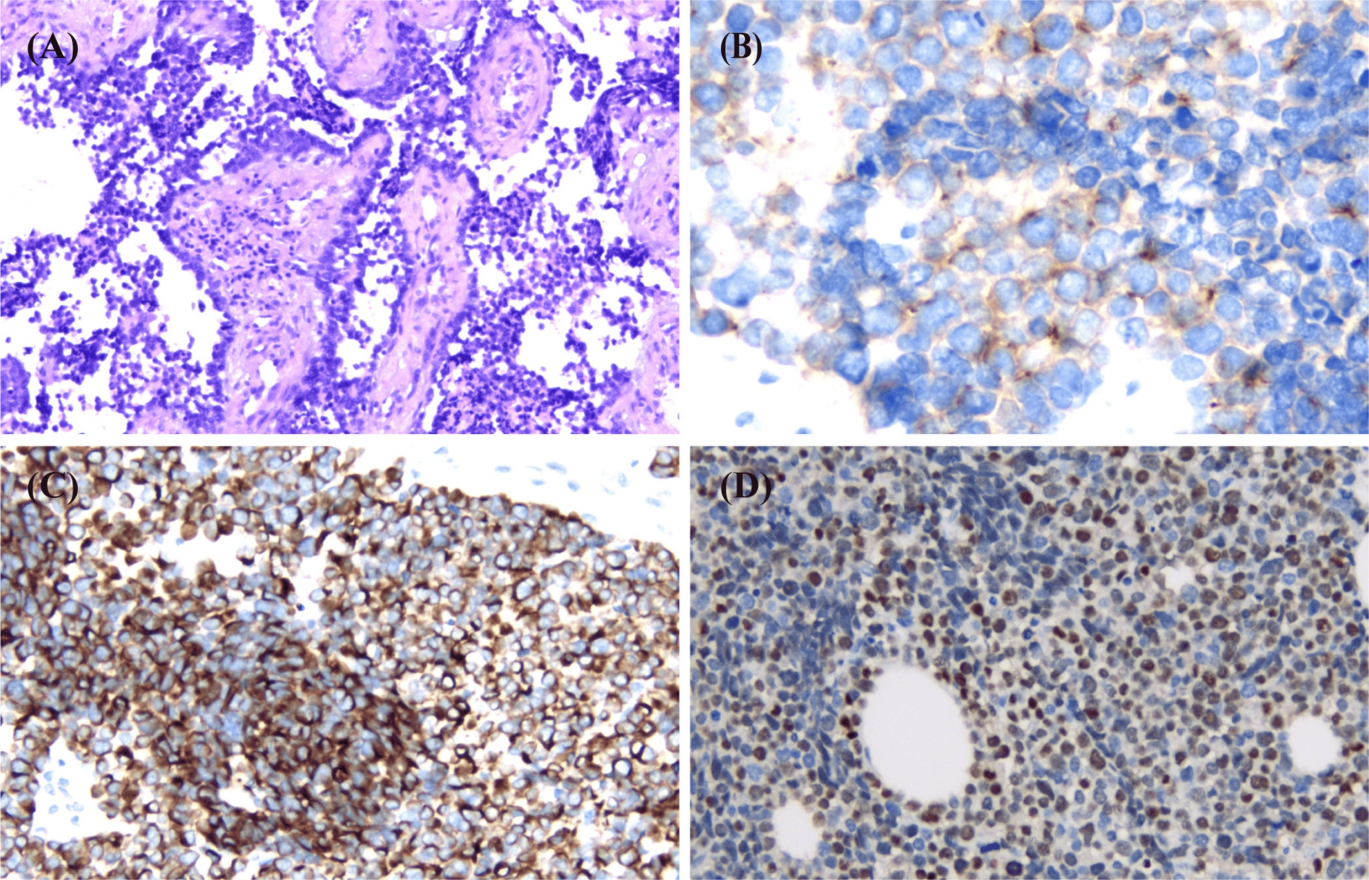
(A) Histopathology of a cervical lymph node biopsy reveals tumor cells arranged in sheets and nests, separated by fibrovascular septa (hematoxylin and eosin, 40×). (B) Immunohistochemistry (IHC) demonstrates ALK paranuclear foci or dot-like positivity (400×). (C) IHC shows membranous desmin expression (200×). (D) IHC reveals nuclear myogenin positivity (200×).

Three days after the aforementioned treatment, the patient's platelet count decreased to 37 × 10^9^/L. Coagulation parameters remained stable. There was no reduction in the size of the cervical lymph nodes, nor was there any relief from pain. On the sixth day, flow cytometry revealed that 88.9% of nucleated cells were CD45-negative and expressed CD9, CD81, and CD56, indicating a non-hematologic neoplasm (Figure 2). We promptly discontinued the use of prednisone acetate. Due to the high risk of bleeding, we performed a cervical lymph node biopsy using a high-frequency electrotome to confirm the diagnosis. Histopathological examination revealed that the tumor cells were arranged in sheets and nests, separated by fibrovascular septa. The tumor cells displayed a round-to-oval morphology, characterized by hyperchromatic nuclei and scant cytoplasm. Immunohistochemical staining indicated that the tumor cells were positive for desmin (membrane+), ALK (paranuclear foci or dot-like+), myogenin (nuclear+), cyclin D1 (partial weak+), CD56 (+), and CD99 (partial weak+), while Ki67 showed approximately 80% positivity. The following markers were negative: PAX-5, EBER, S-100, Ckp (epithelial cells+), C-myc, Mum-1, MPO, INSM1, CD23, CD21, CD43, CD68, Syn, Bcl-6, TdT, TTF-1, and IgD. For B cells, CD20 and CD79α were positive, while CD30 and CD10 were negative. For T cells, CD5, CD3, CD2, and CD7 were positive, whereas SSTR2 and CD123 were negative (Figure 3). We established a diagnosis of ARMS with concurrent DIC based on the histopathological findings of the cervical lymph node biopsy and a score of eight points on the Chinese DIC scoring system (≥7 points) [6]. Although we did not test for the PAX3/7::FOXO1 fusion gene, karyotype analysis of the bone marrow cells revealed a normal karyotype, suggesting the presence of fusion-negative ARMS. However, immunohistochemical staining demonstrated paranuclear foci or dot-like positivity for anaplastic lymphoma kinase (ALK). ALK gene aberrations are more commonly identified in ARMS associated with the PAX3/FOXO1 fusion [7,8].

**Figure 4:**
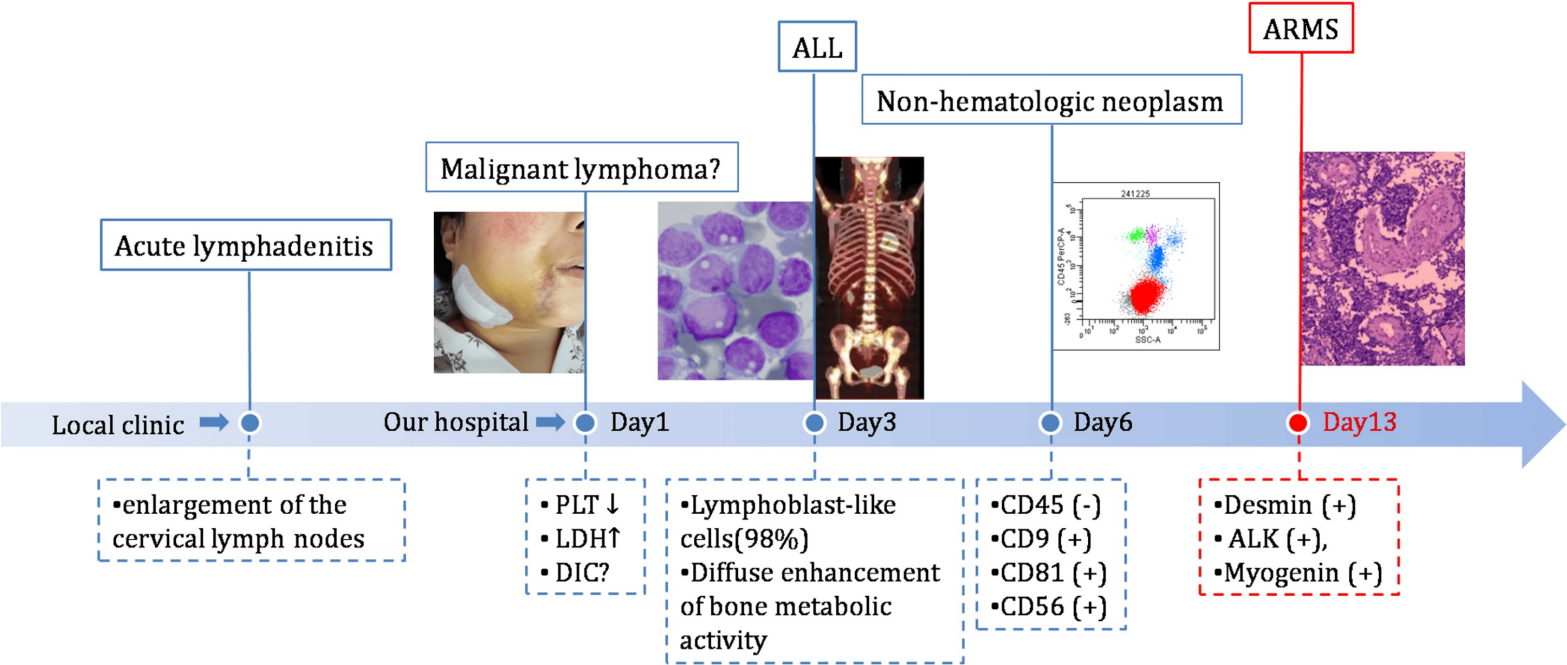
This figure illustrates the patient's diagnostic timeline, which begins with an initial suspicion of acute lymphadenitis at a local clinic. Following an ineffective anti-infective treatment, the patient was referred to our hospital. The initial evaluation—based on enlarged cervical lymph nodes, elevated LDH levels, and possible DIC—suggested a probable malignant lymphoma. Subsequent investigations revealed: (1) diffuse infiltration of lymphoblast-like cells in the bone marrow aspiration; (2) PET/CT evidence of multiple enlarged, hypermetabolic cervical lymph nodes with diffusely increased bone metabolic activity; and (3) potential extramedullary involvement in the right ethmoid sinus, nasal cavity, and maxillary sinus. These findings prompted a revision of the diagnosis to acute lymphoblastic leukemia. When bone marrow flow cytometry subsequently demonstrated CD45-negative tumor cells of non-hematopoietic origin, a cervical lymph node biopsy was performed, ultimately confirming the diagnosis of ARMS.

We summarized the diagnostic process for this patient in a timeline, as illustrated in Figure 4. Initially suspected of having acute lymphadenitis at a local clinic, the patient was referred to our hospital after anti-infective treatment proved ineffective. Upon admission, malignant lymphoma was the preliminary diagnosis. However, subsequent bone marrow aspiration and PET-CT scans led to a revision of the diagnosis to ALL. Flow cytometry results indicated a non-hematopoietic origin of the lymphoblast-like cells in the bone marrow. Ultimately, a cervical lymph node biopsy confirmed the final diagnosis of ARMS.

The patient was referred to the oncology department for further treatment. On the third day of receiving ifosfamide in combination with doxorubicin hydrochloride liposome chemotherapy, the patient experienced a transient loss of consciousness. This incident caused significant concern for both the patient and her family regarding the continuation of chemotherapy. After careful consideration, the patient and her husband decided to discontinue the treatment and return home. During follow-up, the patient remained stable for approximately two weeks; however, she then experienced rapid clinical deterioration. This ultimately led to the patient's death, which occurred 24 days after her discharge.

## Discussion

RMS with diffuse bone marrow infiltration that mimics acute leukemia is a rare occurrence. Of particular concern is the potential misdiagnosis of RMS with bone marrow infiltration as acute leukemia, especially in the absence of identifiable primary lesions. Such misdiagnosis can have critical consequences, as bone marrow infiltration in RMS is also associated with a poor prognosis. Therefore, it is essential to perform a bone marrow aspiration and biopsy to accurately assess the prognosis of RMS patients, even when there are no clinical features indicating bone marrow involvement [1-3,8-10]. In this case, the patient presented with multiple enlarged cervical lymph nodes. Laboratory tests revealed thrombocytopenia, hypofibrinogenemia, and elevated levels of D-dimer, FDP, TAT, and PIC. We performed bone marrow aspirations to confirm the initial diagnosis of malignant lymphoma or acute leukemia. However, bleeding at the puncture site was significantly prolonged, requiring approximately 14 minutes of pressure to achieve hemostasis. Due to the patient's high risk of bleeding, we postponed the planned bone marrow and cervical lymph node biopsy. Based on the bone marrow cytomorphology, we made a diagnosis of ALL and started glucocorticoid therapy. Fortunately, flow cytometry analysis indicated that the "lymphoblasts" were of non-hematopoietic origin. The patient's diagnosis was ultimately confirmed through a biopsy of the cervical lymph nodes. Our team reviewed this case and concluded that the misdiagnosis could not be attributed solely to the tumor cell morphology resembling lymphoblasts and negative POX staining. Furthermore, our lack of awareness regarding the differential diagnosis for RMS led to the incorrect interpretation of the PET-CT findings of masses in the ethmoid sinus, nasal cavity, and maxillary sinus as leukemic infiltration rather than primary RMS lesions.

Distinguishing between lymphoblasts and RMS tumor cells is challenging due to their similar cellular morphology and cytochemical staining characteristics [11]. In this case, as well as in several previously reported cases of RMS with bone marrow involvement that mimic acute leukemia, the presence of scattered vacuoles in the cytoplasm may serve as a point for differential diagnosis [8-12]. In addition, the significant difference in the percentage of blast-like cells between bone marrow and peripheral blood may serve as another point of differentiation. A total of 17 cases of acute lymphoblastic leukemia admitted to our hospital during the same quarter exhibited a mean percentage of 81.4% (ranging from 58% to 95%) of bone marrow lymphoblasts, while the percentage in peripheral blood was 47.29% (ranging from 6% to 82%) at initial diagnosis. By contrast, the percentage of blast-like cells in the bone marrow of this patient was 98%, whereas only 1% was detected in the peripheral blood. This discrepancy has received little attention in previously reported cases, and relevant statistically analyzable data are lacking. One similar case report documented 94.8% blast-like cells with vacuolation in the bone marrow, but only 1% in the peripheral blood [10]. Another case report indicated 89.0% in the bone marrow compared to 0.5% in peripheral blood [9]. In addition, another study confirmed the presence of bone marrow metastases in 19 out of 5,420 bone marrow aspirates and 856 bone marrow trephine biopsies. Among these cases, the 10th patient was diagnosed with RMS with bone marrow metastases; however, only rare blastic cells were detected in the peripheral blood [13]. In conclusion, the presence of scattered vacuoles in the cytoplasm of RMS tumor cells, along with the percentage discrepancy between bone marrow and peripheral blood, as well as the presence of a mass at the primary tumor site, are significant differentiators between RMS bone marrow involvement and acute leukemia. However, of even greater importance is the cultivation of clinical thinking ability that distinguishes these two diseases.

ARMS is characterized by distinct immunohistochemical staining patterns, including desmin, myogenin, and MyoD1, which demonstrate high specificity and sensitivity. Strong and diffuse staining for desmin and myogenin is considered essential diagnostic criteria. Additional markers, such as AP2β, HMGA2, P-cadherin, and EGFR, may assist in subtype classification [2,4]. Approximately 80-90% of ARMS cases harbor translocations involving the FOXO1 gene (located at 13q14.11) and either the PAX3 gene (2q36.1) or the PAX7 gene (1p36.13). Among these cases, 60-70% are characterized by the PAX3::FOXO1 fusion, 10-20% by the PAX7::FOXO1 fusion, and the presence of PAX3/7::FOXO1 fusions is associated with a poorer prognosis [1,2,8]. In addition, a minority of ARMS cases harbor fusion genes such as FOXO1 with FGFR1, PAX3 with FOXO4, or NCOA1/2; however, little is known about the characteristics of these fusion variants of RMS [2,4].

The treatment strategies for RMS include systemic multidrug chemotherapy to target disseminated disease, combined with surgical resection of the primary mass and, when indicated, ionizing radiotherapy for local disease control [1]. RMS with bone marrow involvement and concurrent DIC has been previously reported, often accompanied by tumor lysis syndrome. Appropriate polychemotherapy aimed at reducing malignancy-induced procoagulant activity should be initiated, as it can rapidly correct DIC [12]. Although there is no standardized chemotherapy regimen for adult RMS patients, some studies suggest using a combination of ifosfamide, doxorubicin, and vincristine, while others advocate for pediatric regimens such as vincristine, actinomycin D, and cyclophosphamide. However, the prognosis for adult RMS patients remains poor compared to pediatric patients, with five-year survival rates ranging from 26.6% to 61% [1].

## Conclusions

RMS presents clinical features and bone marrow morphology that closely resemble those of acute leukemia. The potential diagnosis of RMS should be considered in cases of bone marrow infiltration by blast-like cells that morphologically resemble lymphoblasts but lack hematopoietic markers. Key points for differential diagnosis, before establishing a definitive histopathologic diagnosis, may include the presence of blast-like cells with scattered cytoplasmic vacuolation, a significant discrepancy in the proportion of blast-like cells between the bone marrow and peripheral blood, and imaging findings of a primary mass in anatomical sites typically associated with RMS. However, these differential points require the collection of more similar cases for confirmation. A definitive diagnosis necessitates a biopsy of the bone marrow or primary mass, accompanied by immunohistochemical staining for markers such as desmin, myogenin, and MyoD1. In addition, molecular testing for PAX3/7::FOXO1 fusion status is essential for prognostication and subtype classification.
